# High Risk Population Isolate Reveals Low Frequency Variants Predisposing to Intracranial Aneurysms

**DOI:** 10.1371/journal.pgen.1004134

**Published:** 2014-01-30

**Authors:** Mitja I. Kurki, Emília Ilona Gaál, Johannes Kettunen, Tuuli Lappalainen, Androniki Menelaou, Verneri Anttila, Femke N. G. van 't Hof, Mikael von und zu Fraunberg, Seppo Helisalmi, Mikko Hiltunen, Hanna Lehto, Aki Laakso, Riku Kivisaari, Timo Koivisto, Antti Ronkainen, Jaakko Rinne, Lambertus A. L. Kiemeney, Sita H. Vermeulen, Mari A. Kaunisto, Johan G. Eriksson, Arpo Aromaa, Markus Perola, Terho Lehtimäki, Olli T. Raitakari, Veikko Salomaa, Murat Gunel, Emmanouil T. Dermitzakis, Ynte M. Ruigrok, Gabriel J. E. Rinkel, Mika Niemelä, Juha Hernesniemi, Samuli Ripatti, Paul I. W. de Bakker, Aarno Palotie, Juha E. Jääskeläinen

**Affiliations:** 1 Neurosurgery, NeuroCenter, Kuopio University Hospital, Kuopio, Finland; 2 Neurosurgery, Institute of Clinical Medicine, University of Eastern Finland, Kuopio, Finland; 3 Department of Neurobiology, A.I. Virtanen Institute for Molecular Sciences, University of Eastern Finland, Kuopio, Finland; 4 Department of Neurosurgery, Helsinki University Central Hospital, Helsinki, Finland; 5 Institute for Molecular Medicine Finland (FIMM), University of Helsinki, Helsinki, Finland; 6 Department of Chronic Disease Prevention, National Institute for Health and Welfare, Helsinki, Finland; 7 Department of Genetic Medicine and Development, University of Geneva Medical School, Geneva, Switzerland; 8 Department of Medical Genetics, University Medical Center Utrecht, Utrecht, The Netherlands; 9 Analytical and Translational Genetics Unit, Department of Medicine, Massachusetts General Hospital and Harvard Medical School, Boston, Massachusetts, United States of America; 10 Program in Medical and Population Genetics, Broad Institute of Harvard and MIT, Cambridge, Massachusetts, United States of America; 11 UMC Utrecht Stroke Center, Department of Neurology and Neurosurgery, Rudolf Magnus Institute of Neuroscience, University Medical Center Utrecht, The Netherlands; 12 Neurology, Institute of Clinical Medicine, University of Eastern Finland, Kuopio, Finland; 13 Department of Urology, Radboud University Nijmegen Medical Centre, Nijmegen, The Netherlands; 14 Department for Health Evidence, Radboud University Nijmegen Medical Centre, Nijmegen, The Netherlands; 15 Folkhälsan Research Centre, Helsinki, Finland; 16 Department of Chronic Disease Prevention, National Institute for Health and Welfare, Helsinki, Finland; 17 Department of General Practice and Primary Health Care, University of Helsinki, Helsinki, Finland; 18 Department of Internal Medicine, Vasa Central Hospital, Vasa, Finland; 19 Unit of General Practice, Helsinki University Central Hospital, Helsinki, Finland; 20 Estonian Genome Center, University of Tartu, Tartu, Estonia; 21 Department of Clinical Chemistry, Fimlab Laboratories, Tampere University Hospital and University of Tampere, Tampere, Finland; 22 Department of Clinical Physiology and Nuclear Medicine, University of Turku and Turku University Hospital, Turku, Finland; 23 Research Centre of Applied and Preventive Cardiovascular Medicine, University of Turku and Turku University Central Hospital, Turku, Finland; 24 Department of Neurosurgery, Department of Neurobiology and Department of Genetics, Program on Neurogenetics, Howard Hughes Medical Institute, Yale School of Medicine, New Haven, Connecticut, United States of America; 25 Hjelt Institute, University of Helsinki, Helsinki, Finland; 26 Division of Genetics, Department of Medicine, Brigham and Women's Hospital, Harvard Medical School, Boston, Massachusetts, United States of America; 27 Department of Epidemiology, University Medical Center Utrecht, Utrecht, The Netherlands; 28 Department of Human Genetics, The Wellcome Trust Sanger Institute, Cambridge, United Kingdom; University of California San Francisco, United States of America

## Abstract

3% of the population develops saccular intracranial aneurysms (sIAs), a complex trait, with a sporadic and a familial form. Subarachnoid hemorrhage from sIA (sIA-SAH) is a devastating form of stroke. Certain rare genetic variants are enriched in the Finns, a population isolate with a small founder population and bottleneck events. As the sIA-SAH incidence in Finland is >2× increased, such variants may associate with sIA in the Finnish population. We tested 9.4 million variants for association in 760 Finnish sIA patients (enriched for familial sIA), and in 2,513 matched controls with case-control status and with the number of sIAs. The most promising loci (p<5E-6) were replicated in 858 Finnish sIA patients and 4,048 controls. The frequencies and effect sizes of the replicated variants were compared to a continental European population using 717 Dutch cases and 3,004 controls. We discovered four new high-risk loci with low frequency lead variants. Three were associated with the case-control status: 2q23.3 (MAF 2.1%, OR 1.89, p 1.42×10-9); 5q31.3 (MAF 2.7%, OR 1.66, p 3.17×10-8); 6q24.2 (MAF 2.6%, OR 1.87, p 1.87×10-11) and one with the number of sIAs: 7p22.1 (MAF 3.3%, RR 1.59, p 6.08×-9). Two of the associations (5q31.3, 6q24.2) replicated in the Dutch sample. The 7p22.1 locus was strongly differentiated; the lead variant was more frequent in Finland (4.6%) than in the Netherlands (0.3%). Additionally, we replicated a previously inconclusive locus on 2q33.1 in all samples tested (OR 1.27, p 1.87×10-12). The five loci explain 2.1% of the sIA heritability in Finland, and may relate to, but not explain, the increased incidence of sIA-SAH in Finland. This study illustrates the utility of population isolates, familial enrichment, dense genotype imputation and alternate phenotyping in search for variants associated with complex diseases.

## Author Summary

Genome-wide association studies (GWAS) have been extensively used to identify common genetic variants associated with complex diseases. As common genetic variants have explained only a small fraction of the heritability of most complex diseases, there is a growing interest in the role of how low frequency and rare variants contribute to the susceptibility. Low frequency variants are more often specific to populations of distinct ancestries. Saccular intracranial aneurysms (sIA) are balloon-like dilatations in the arteries on the surface of the brain. The rupture of sIA causes life-threatening intracranial bleeding. sIA is a complex disease, which is known to sometimes run in families. Here, we utilize the recent advancements in knowledge of genetic variation in different populations to examine the role of low-frequency variants in sIA disease in the isolated population of Finland where sIA related strokes are more common than in most other populations. By studying >8000 Finns we identify four low-frequency variants associated with the sIA disease. We also show that the association of two of the variants are seen in other European populations as well. Our findings demonstrate that multiple study designs are needed to uncover more comprehensively their genetic background, including population isolates.

## Introduction

About 3% of the population develops saccular intracranial aneurysms (sIAs) during life [1], [2]. Some 95% of subarachnoid hemorrhages are caused by ruptured sIA (sIA-SAH), a devastating form of stroke affecting individuals mainly in the sixth decade of life [3]. The annual incidence of SAH is 4–9 per 100 000 worldwide [4] but over twice as high in Finland and in Japan [5]. The sIA disease is a complex trait, the risk of which is affected by age, sex, smoking, hypertension, excess drinking [6], and in over 10% of the cases family history of sIA disease [7]–[9].

To date, genome wide association (GWA) studies have identified six definite and one probable loci with common variants associated to sIA: 4q31.23 (OR 1.22) [10], [11]; 8q11.23–q12.1 (OR 1.28); 9p21.3 (OR 1.31); 10q24.32 (OR 1.29); 12q22 (OR 1.16) [10]; 13q13.1 (OR 1.20); 18q11.2 (OR 1.22) [12] (Table S5). These seven loci were estimated to explain 6.1%, 4.4% and 4.1% of the four-fold sibling recurrence risk in Finland, Europe and Japan respectively [10]. In these previous GWA studies, results on 2q33.1 locus were inconsistent: the locus was significant in the first GWAS [13], not significant in the enlarged follow-up GWAS [12], and in the third GWAS the risk allele was reversed in the Japanese replication sample [10].

The population of Finland is one of the most thoroughly characterized genetic isolates. Due to the small size of the founder population, subsequent bottleneck effects and genetic drift, the Finnish population is enriched for rare and low frequency variants that are almost absent in other European populations and some variants rare elsewhere are increased in frequency [14]. This is best illustrated by the increased prevalence of 36 rare Mendelian, mostly recessive, disorders in Finland (www.findis.org); the so called Finnish disease heritage (FDH) [15]. We hypothesized that some of the enriched rare or low frequency variants could contribute to the increased sIA-SAH susceptibility in Finland.

**Figure 1:**
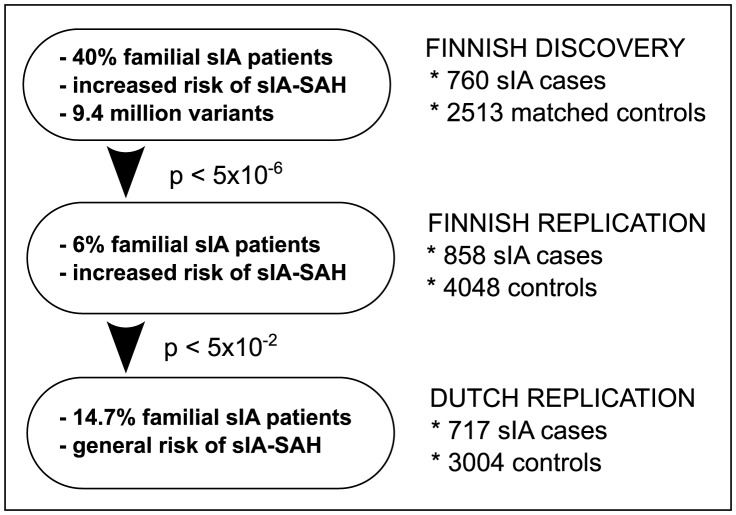
Study design. The Finnish discovery and replication cohorts represent a population with over two-fold increased risk of subarachnoid hemorrhage from ruptured saccular intracranial aneurysm (sIA-SAH). The Finnish discovery cohort was intentionally enriched with familial sIA patients, and 9.4M genotyped and imputed variants were studied. The loci with p<5E-6 were replicated in an independent and unselected Finnish sIA sample. The allele frequencies and effect sizes of the replicated variants in Finland were finally compared to continental European population using a Dutch sample. The sIA-SAH risk is not increased in the Netherlands (‘general risk’ in the figure).

In this GWA study we combined the power of 1000 Genomes imputation, the special benefits of a population isolate and enrichment of familial cases in the discovery cohort. Familial sIA patients more often carry multiple sIAs as compared to sporadic sIA patients, which may confer additional genetic burden to the sIA formation [8], [16], [17]. Therefore, in addition to the case vs. control analysis, we also analyzed the number of sIAs per individual as an intermediate phenotype. We conducted an association analysis in a discovery sample of 760 Finnish sIA cases and 2,513 matched controls followed by replication in an additional sample of 858 Finnish sIA cases and 4048 controls. The successfully replicated loci in Finland were further studied in a Dutch cohort of 717 sIA cases and 3004 controls to assess the extent to which the allele frequencies and risk effect sizes match between the isolate of Finland and a continental European population (Figure 1). In addition, we hypothesized that a previously inconclusive locus on 2q33.1 [10], [13], [18] is a true sIA risk locus at least in Finland and aimed to replicate the best discovery associations in the locus in this study in the Finnish and in the Dutch samples.

We successfully identified associations with low frequency variants in three novel loci in the case vs. control analysis and one in the aneurysm count analysis. Two of the case vs. control loci replicated also in the Dutch cohort with similar allele frequencies and comparable risk effect sizes. The variant in the aneurysm count locus demonstrated a strong bottleneck effect by being 15 times more frequent in the Finnish than in the Dutch controls. We also successfully replicated the previously inconclusive 2q33.1 locus.

## Results

### Case vs. control analysis in Finnish and Dutch samples

To increase the potential genetic load in the study sample, our discovery sample consisted of 760 cases from the isolated, high-risk Finnish population, purposefully enriched for familial sIA (40%) patients and 2513 genetically matched Finnish controls. The imputation of the 304,399 previously genotyped variants [12] against the 1000 Genomes Project reference panel (v3, March 2012 release) increased the number of common and low frequency variants available for the association analysis to 9,359,231. Quantile-quantile (QQ) plots of association p-values did not indicate substantial inflation (λ = 1.04) (Figure S1). The discovery association analysis revealed one locus at 12p11.1 driven by rs653464 at conventional genome-wide significance (p<5×10^−8^) and 14 other loci at p<5×10^−6^ (Table S1; Manhattan plot in Figure S3).

We chose 17 SNPs representing the 15 promising loci (p<5×10^−6^) above for replication in an independent sample of 858 Finnish sIA cases and 4,048 controls (Table 1). Four SNPs and one deletion were associated at p<0.05 with the sIA disease (Table S1), two of them in the previously reported sIA loci 9p21.3 (rs1333042; OR 1.3, p = 6.3×10^−7^) and 13q13.1 (rs113124623; OR 0.88, p = 0.01). The genome-wide significant 12p11.1 locus in the discovery sample did not replicate (p = 0.29).

**Table 1:** The Finnish and Dutch study samples used in the association analysis of saccular intracranial aneurysm (sIA) disease.

	Finnish discovery		Finnish replication		Dutch replication	
	Cases	Controls	Cases	Controls	Cases	Controls
N	760	2,513	858	4,048	717	3,004
Women	443 (58%)	1,454 (58%)	532 (62%)	2,182 (54%)	492 (67%)	1,135 (38%)
Familial sIA	300 (40%)	-	51 (6%)	-	100 (15%)*	-
sIA-SAH	561 (74%)	-	587 (68%)	-	658 (92%)	-
Mean age (SD)	50 (12.6)	56 (13)	52 (12.2)	40 (9.95)	54 (11.7)	68 (10.44)
Number of sIAs						
Mean (range)	1.54 (1–8)	-	1.46 (1–6)	-	1.26 (1–7)	-
≥2	242 (32%)	-	257 (30%)	-	127 (18%)**	-

* Unknown familial sIA status for 35 patients.

** Number of sIAs not known for 16 patients.

SD = Standard deviation.

In the meta-analysis of the two Finnish samples, four SNPs reached the commonly used level of genome-wide significance at p<5×10^−8^ (Table 2). Three were novel: 2q23.3 (rs74972714; OR 2.1, 95% CI 1.68–2.63, p = 7.4×10^−11^, control allele frequency or CAF 2.35%), 5q31.3 (rs113816216; OR 1.92, CI 1.53–2.40, p = 1.74×10^−8^, CAF 2.09%) and 6q24.2 (rs75018213; OR 1.97, CI 1.6–2.43, p = 2.25×10^−10^, CAF 2.53%). One was previously reported at 9p21.3 (rs1333042; OR 1.31, CI 1.21–1.42, p = 1.8×10^−11^, CAF 42.3%) (Table 2). We assessed the robustness of the associations controlling also for age and the effect sizes and p-values were almost identical (data not shown).

**Table 2:** Five loci with a genome-wide significant association to saccular intracranial aneurysm (sIA) disease in the Finnish and Dutch samples.

Case vs. control analysis		Finnish discovery				Finnish replication				Finnish meta-analysis				Dutch replication				All meta-analysis	
SNP*	Gene	Case MAF	Ctrl MAF	OR	P	Case MAF	Ctrl MAF	OR	P	Case MAF	Ctrl MAF	OR	P	Case MAF	Ctrl MAF	OR	P	OR§	P
rs74972714 (C/A) 2q23.3 (150370860 bp)	LYPD6 (40 kb)**	0.034	0.017	2.73	3.43E-06	0.049	0.028	1.88	4.11E-06	0.0421	0.0235	2.10	7.41E-11	0.017	0.016	1.04	4.37E-01	1.89	1.42E-09
rs113816216 (G/C) 5q31.1 (132846228 bp)	FSTL4***	0.045	0.021	2.31	8.26E-07	0.032	0.021	1.60	2.57E-03	0.0382	0.0209	1.92	1.74E-08	0.045	0.039	1.30	4.53E-02	1.66	3.17E-08
rs75018213 (A/G) 6q24.2 (146052178 bp)	EPM2A***	0.051	0.027	2.11	3.44E-06	0.042	0.024	1.85	2.85E-05	0.0461	0.0253	1.97	2.25E-10	0.029	0.023	1.50	3.39E-02	1.87	7.14E-11
rs1333042 (G/A)† 9p21.3(22103813 bp)	CDKN2B-AS1	0.500	0.432	1.32	3.01E-06	0.481	0.417	1.30	6.30E-07	0.490	0.423	1.31	1.81E-11	0.543	0.479	1.32	3.42E-06	1.31	6.71E-16
rs919433 (A/G)‡ 2q33.1(198166565 bp)	ANKRD44***	0.480	0.428	1.25	2.53E-04	0.486	0.446	1.18	1.01E-03	0.483	0.440	1.21	2.15E-06	0.418	0.332	1.43	9.77E-09	1.27	2.20E-12
rs12472355 (A/C)‡ 2q33.1(198205840 bp)	ANKRD44 (30 kb)**	0.478	0.427	1.24	2.89E-04	0.488	0.443	1.21	2.23E-04	0.483	0.437	1.23	4.84E-07	0.391	0.310	1.39	1.05E-07	1.27	1.87E-12

* For each variant minor allele/major allele, locus and base pair position are given.

** The variant's distance (kb) to the nearest gene is given.

*** Located in the intron of the given gene.

† The previously reported 9p21.3 locus [12], [39].

‡ The previously studied 2q33.3 locus with inconclusive evidence (see Materials and Methods).

§ Some heterogeneity in effect sizes exists between cohorts. See Table S9 for heterogeneity statistics.

To assess how the allele frequencies and effect sizes of variants identified in the Finnish population compare to other European populations, we studied those variants in a Dutch sample consisting of 717 sIA cases and 3,004 controls (Table 1). All three variants tagging the novel loci at 2q23.3, 5q31.3 and 6q24.2 had a similar low minor allele frequency (1.6–3.9%) in Finland and the Netherlands (Table 2). Two of them had similar effect sizes and were also replicated: 5q31.3 (rs113816216; OR 1.3, CI 0.98–1.75, p = 0.045, CAF 3.87%) and 6q24.2 (rs75018213; OR 1.5, CI 0.98–2.3 p = 0.034, CAF 2.3%). The previously reported 9p21.3 locus also replicated in the Dutch sample (rs1333042; OR 1.32, CI 1.17–1.49, p = 3.42×10^−6^, CAF 47.86%).

In the meta-analysis of the Finnish and Dutch samples, all three novel loci 2q23.3 (rs74972714; OR 1.89, p = 1.42×10^−9^), 5q31.3 (rs113816216; OR 1.66, p = 3.17×10^−8^) and 6q24.2 (rs75018213; 1.87, p = 7.1×10^−11^) were significantly associated to the sIA disease at genome-wide significance (Table 2; see Table S7 for imputation accuracy statistics). Some heterogeneity in effect sizes exists between samples (Table S9).

As the standard genome-wide significance 5×10^−8^ is estimated to correct for independent tests of common variants (MAF> = 5%) and we tested also a set of low-frequency variants, the common significance level may be too liberal. Based on Europeans of the 1000 Genomes project we estimated the significance level to be 3.82×10^−8^ (See Materials and Methods). All of the reported variants are below this level.

### Association of variants to the number of sIAs

Some 20–30% of the sIA patients carry multiple sIAs, a phenomenon more commonly seen in familial sIA disease [8], [16], [17]. We hypothesized that an increased number of sIAs (≥2) in a given patient would reflect a higher underlying genetic load, motivating us to use aneurysm count as an intermediate phenotype to increase statistical power. The number of sIAs was used as a count data using the negative binomial regression analysis in the discovery sample of 760 Finnish sIA cases (1–8 sIAs per patient) and 2,513 controls. The QQ plot (Figure S2) and the genomic inflation factor (1.05) did not indicate substantial population stratification.

Nine loci had variants at p<5E-6 (Table S2; Manhattan plot in Figure S4). The most significant variant of each locus was selected for replication in the new Finnish sample of 858 sIA cases (1–6 sIAs per patient) and 4,048 controls. Two loci were replicated at p<0.05: 7p22.1 (rs150927513; RR 1.39, p = 8.36×10^−4^, CAF 5.24%) and 16p13.3 (rs144159053; rate ratio (RR) 1.66, p = 4.4×10^−3^, CAF 1.27%) (Table S2). rs10802056 on 1p12 had a significant association p-value but the effect direction was different and thus was not considered as replicated. We assessed the robustness of the associations controlling also for age and the effect sizes and p-values were almost identical (data not shown).

In the meta-analysis of the Finnish samples, 7p22.1 was genome-wide significant (rs150927513; RR 1.6, CI 1.37–1.88, p = 4.92×10^−9^, CAF 4.61%);**Table 3**; See genotype to aneurysm count distribution in Table S3). The rate ratio (RR) estimate is the relative rate of aneurysm formation (i.e. change in expected number of aneurysms) per allele as compared to minor allele homozygotes.

**Table 3:** The locus with a genome-wide significant association to the number of saccular intracranial aneurysms (sIA) per individual in the Finnish samples.

Association to sIA count		Finnish discovery				Finnish replication				Finnish meta-analysis				Dutch replication				All meta-analysis§	
SNP*	Gene	Case MAF	Ctrl MAF	RR	P	Case MAF	Ctrl MAF	RR	P	Case MAF	Ctrl MAF	RR	P	Case MAF	Ctrl MAF	RR	P	RR	P
rs150927513 (T/A) 7p22.1 (4894744 bp)	RADIL**	0.060	0.036	1.95	8.86E-08	0.070	0.052	1.39	8.36E-4	0.0653	0.0461	1.60	4.92E-09	0.003	0.003	0.97	4.82E-1	1.59	6.08E-09

* For each variant major allele/minor allele, locus and base pair position are given.

** Located in the intron of the given gene.

§ See Table S9 for heterogeneity statistics.

To compare the allele frequency and effect size of rs150927513 identified in the Finnish population to those of continental European populations, we studied the variant also in the Dutch, but the imputation quality (Impute info 0.38) and estimated allele frequency (0.29%) were too low to obtain reliable estimates (RR 0.97; 95% CI 0.17–4.03, p = 0.97). We additionally checked the minor allele frequency of rs150927513 in 498 whole-genome sequenced Dutch individuals of GENOMEoftheNETHERLANDS-project (http://www.nlgenome.nl/). Only two individuals were heterozygous and the rest were major allele homozygotes (MAF 0.2%), which is in agreement with our imputation results of the Dutch sample.

### Analysis of 2q33.1 locus

Previously published results on the 2q33.1 locus are inconsistent, being significant in the first GWAS [13], not significant in the enlarged follow-up GWAS [12], and uncertain in the third GWAS [10]. We aimed to study if the 2q33.1 would replicate in Finland, even though no variant in this region reached p<5E-6 in the discovery sample. We chose two of the most significant SNPs (in this study) at 2q33.1 for replication in the new Finnish replication sample, which was not used in the previous studies (rs12472355; OR 1.21, p = 2.23×10^−4^, CAF 44.3%, and rs919433; OR 1.18, p = 1.01×10^−3^, CAF 44.6%). They are in LD with the three previously investigated SNPs (rs787994, rs1429412, rs700651; LD r^2^ 0.75–0.96). The variants rs12472355 (OR 1.23, CI 1.13–1.33, p = 4.84×10^−7^) and rs919433 (OR 1.21, CI 1.12–1.31, p = 2.15×10^−6^) did not reach genome-wide significance in the combined Finnish samples (Table 2). They were highly significant in the Dutch sample (rs12472355; OR, 1.39, CI 1.23–1.57, p = 1.05×10^−7^ and rs919433; OR 1.43, CI 1.26–1.61, p 9.77×10^−9^), and in the meta-analysis of all three samples they reached genome-wide significance (Table 2). The allele frequencies were notably higher in the Finnish samples (44% and 43.7%) than in the Dutch samples (33.2% and 31%).

### Heritability estimate

We estimated the heritability explained by the reported variants. The four novel loci on 2q23.3, 5q31.3, 6q24.2 and 7p22.1 were estimated to explain 1.7% of the heritability in the combined Finnish samples. Adding the previously inconclusive 2q33.1 locus increases the heritability explained to 2.1%.

### Genotype validation

For validating the imputation accuracy, we genotyped 87 individuals of the discovery sample using Sequenom genotyping. The concordance rates range from 96–99% except rs74972714 was slightly lower at 94% (Table S8). We did additional validation by Sanger sequencing 10 individuals per variant who were predicted to carry minor alleles. The imputation was near perfect in all other SNPs except rs75018213 had discrepancies between major allele homozygote and heterozygotes (Table S11). We further estimated by simulation, how likely it would be to get the observed OR for rs75018213 in the discovery sample just by change, given the imputation accuracy (See Text S1 for details). The probability of chance finding was very low (p: 0.0001) even if assuming that the minor allele would be over-imputed by 20% in cases (p: 0.004).

Some individuals were genotyped by both Sanger sequencing and Sequenom and the concordance between the two methods was perfect (Table S11). Finally, we estimated, *in silico*, the imputation efficiency of reported SNPs in Dutch population. 96 individuals of the Genome of the Netherlands project had both high coverage whole-genome sequencing (40×) data as well as GWA chip genotyping data available. We imputed the genotypes of reported SNPs using the same imputation methods, 1000 Genomes reference panel and set of SNPs in GWA chips as was done in the discovery and Dutch comparison analyses. The genotype concordance rates were excellent (Table S13). It is noteworthy that the imputation quality measure reported by the Impute2 program was higher in all of the SNPs in our Dutch replication cohort (Table S7) than in the *in silico* validation experiment. This indicates excellent imputation quality in the Dutch replication.

### Fine mapping of the identified loci

We attempted to identify putative causative variants from whole exome sequencing data of 583 Finnish individuals. We focused on variants within 1 MB of the lead SNPs with high impact on protein product (i.e. variants affecting splice site, losing or gaining stop/start codon, altering reading frame) or non-synonymous coding SNPs. We additionally filtered variants if they were not in LD with the lead SNPs (r2<0.4, Europeans of 1000 Genomes if available). 254 variants were identified, most of which were rare. However 15 variants were enriched to low-frequency range (MAF>1%) (Table S12). The impact of these variants needs to be evaluated in follow-up studies.

### Regulatory elements at identified loci

The UCSC Genome Browser and HaploReg version 2 [19] were used to search for ENCODE regulatory elements at the five genome-wide significant variants.

rs74972714 at 2q23.3 and rs150927513 at 7p22.1 reside within a DNAse hypersensitivity peak. The rs75018213 at 6q24.2 resides on an ENCODE *GATA2* transcription factor binding site peak (Table S4).

Using genome-wide Chip-SEQ analysis, Ernst et al. constructed a predicted cell-type specific regulatory region map of nine chromatin marks in nine cell lines [20]. rs113816216 at 5q31.3 resides on a predicted erythroleukemia cell specific (K562) strong enhancer and rs75018213 at 6q24.2 on a predicted lymphoblastoid cell (GM12878) weak enhancer (Table S4).

We searched for putative transcription factor binding sites affected by the four variants, based on position weight matrices from Transfac, Jaspar and ENCODE (top 3 enriched motifs for each transcription factor, identified in transcription factor Chip-SEQ peaks [19]). rs74972714 at 2q23.3 affects putative binding sites for EBF1 (ENCODE), HDAC2 (ENCODE), *RXRA:PPARG* complex (Transfac), *ZNF423* (Jaspar) and *ZIC3* (Jaspar). rs113816216 at 5q31.3 affects the putative binding sites for *RFX1* (Transfac), *SREBP1 (ENCODE)*, *STAT3* (Transfac) and *IKZF3* (Transfac). rs150927513 at 7p22.1 affects putative binding sites of *T* (brachyury) (Transfac), *CEBPB (Transfac)* and P300 (ENCODE). rs75018213 at 6q24.2 is not directly on any putative transcription factor binding site. (Table S4).

At the 2q33.1 locus neither of the studied variants (rs919433, rs12472355) are on ENCODE DNAse hypersensitivity or transcription factor binding site peaks. However, rs919433 is on a predicted lymphoblastoid (GM12878) cell enhancer whereas rs12472355 is not directly on any regulatory region. rs919433 disrupts a putative transcription factor binding sites for *RUNX2* (OSF2,Transfac) and the *MYC:MAX* complex (Transfac).

### eQTL analysis

To study the potential effects of the variants in the five significant loci on the transcripts of nearby genes, we correlated the variants to expression levels of exons of nearby genes (expression quantitative trait locus (eQTL) analysis) obtained using RNA-sequencing in lymphoblasts of genotyped European individuals from the 1000 Genomes Project (Finnish, British, Toscani and CEPH populations, n = 373; www.geuvadis.org, [21]). Each variant was correlated to transcripts residing within 1 MB. There were 55 genes in 586 exons available for analysis (see Materials and Methods) and in total 748 tests were performed corresponding to Bonferroni corrected significance threshold of 8.7×10^−5^. Strongest association for each variant are reported below and all eQTL results in Table S6.

The most significant eQTL associations were observed at the 2q33.1 locus: rs12472355 associated significantly to the closest gene *ANKRD44* (per allele fold change (FC) 0.94, p = 1.83×10^−5^) and also to *HSPD1* (FC 0.94, p = 1.6×10^−4^), whereas rs919433 was associated to the same genes but in different order of significance; *HSPD1* (FC 0.94, p = 3.8×10^−5^) and *ANKRD44* (FC 0.95, p = 1.4×10^−4^). Among the novel low-frequency variants, only rs150927513 at 7p22.1 was significantly associated to *TNRC18* (FC 1.23, p = 5.1×10^−5^). Nominal associations were observed for two other novel low frequency variants: rs113816216 at 5q31.3 to *VDAC1* (FC 2.12, p 4.6E-4); rs74972714 at 2q23.3 to *EPC2* (FC 0.75, p = 3.9×10^−2^). rs75018213 at 6q24.2 did not have any association even at nominal p<0.05 (Table S6).

We additionally investigated the eQTL landscape of identified loci by pairwise comparison of p-values from eQTL (MAF>0.05 p<0.001) and sIA analyses (Figure S5) and by plotting eQTL associations (p<0.001) in the implicated loci (Supplementary Figure S6 A–E). Only few loci show strong (p<1E-5) association in eQTL and at least nominal (p<0.05) association to sIA (Table S10). There does not seem to be stronger eQTL associations in LD with the lead SNPs. In the 2q33.1, where the lead SNPs were significantly associated to transcript levels, there seems to be a lot of regulatory potential in the same locus, even though not in direct LD with the lead variants (Figure S6 E).

## Discussion

In this study, we used three approaches to improve the power to identify new loci associated to the sIA disease. First, we focused on the Finnish population isolate with increased risk for subarachnoid haemorrhage from ruptured sIAs (sIA-SAH) [5]. Second, we enriched the proportion of familial sIA patients in the discovery sample, thus possibly increasing the prevalence of risk alleles. Third, we increased genome-wide coverage through imputing ungenotyped variants based on 1000 Genomes Project data. The used 1000 Genomes Project imputation reference panel included 93 Finns, which made it well suited for discovery of enriched sIA associated variants in the Finnish population. Using this combination of strategies, we were able to identify three new loci associated with sIA disease, and one locus associated with the number of aneurysms. Additionally we replicated a locus where the evidence so far was inconclusive. Together these five loci account for 2.1% of the heritability in the Finnish samples. In comparison, the six previously identified SNPs explain 2.5% of the heritability in the discovery sample of the current study. Our results likely reflect the varying genetic background of complex traits, such as sIA, in different populations.

### Four novel sIA loci

The lead SNPs in the four novel loci all have a low frequency (<5%) in the general population and could not have been identified without imputing the genotype data against the 1000 Genomes reference. One of the variants, rs150927513 at 7p22.1 that was associated with the number of sIAs, indicates a strong bottleneck effect, for it was 15 times more frequent in the controls of combined Finnish samples (4.6%) than in the Dutch sample (0.3%), and it is virtually non-existent in other populations (1000 Genomes). The three other loci had similar frequencies in Finland and other European populations (1000 Genomes). These four novel loci explain 1.7% of the heritability in the Finnish samples.

The four sIA loci had higher effect sizes (point estimates ranging from 1.59 to 1.88) than the lead SNPs identified by previous GWA studies. We cannot yet conclude whether relatively high ORs of low frequency risk alleles are a typical feature of sIA disease. Similar, and higher, odds ratios for low frequency and rare variants have been reported in isolates for other traits [22], [23]. It is likely that this first wave of low frequency and rare susceptibility variants represent “low hanging” fruits that do not allow general conclusions about the susceptibility landscape of sIA or other complex traits.

### 2q23.3 locus

**Figure 2:**
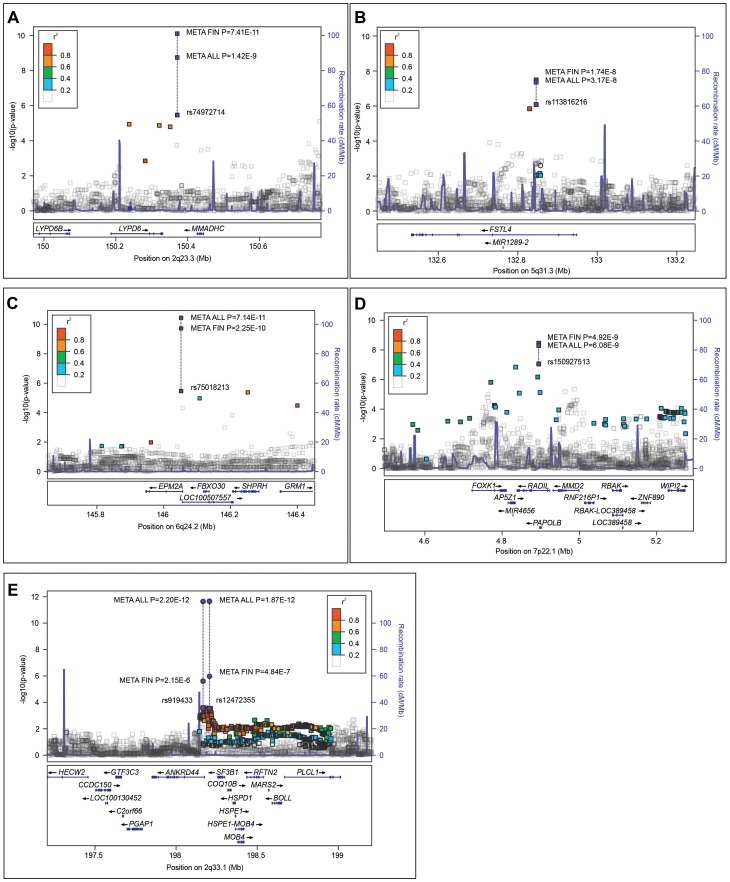
Regional association plots of the five identified saccular intracranial aneurysm (sIA) loci in the combined Finnish samples and the Dutch sample. Association p-values (−log10 scale, y-axis) of variants are shown according to their chromosomal positions (x-axis). Blue lines indicate the genetic recombination rate (cM/Mb). Figures A–C present the loci identified in the case vs. control analysis at 2q23.3, 5q31.3, and 6q24.2, respectively. Figure D presents the 7p22.1 locus associated to the sIA count per patient. Figure E presents the 2q33.1 locus with inconclusive previous evidence. Purple rectangles indicate the most significant variant in a) the Finnish discovery sample and, along the dashed line, its p-values in b) the combined Finnish samples (META FIN) and in c) all samples (META ALL). Adjacent variants in linkage disequilibrium (r^2^; EUR populations, 1000 Genomes March 2012) to the index variant are shown in colours indicating their r^2^ levels (r^2^ box in each figure).

The variant rs74972714 at 2q23.3 has a frequency of about 2% in European populations, including Finns. It was significantly associated to sIA in the Finnish samples but did not show a trend for being associated in the Dutch sample despite having a similar allele frequency. Further studies are required to find out whether this variant tags a risk allele specific to Finnish sIA patients. The variant is located 40 kb downstream of *LYPD6* and 55 kb upstream of *MMADHC* (Figure 2 A). *LYPD6* has recently been characterized as a member of the Ly-6 protein superfamily [24]. *LYPD6* is ubiquitously expressed with highest levels in heart and brain. GPI-anchored Ly-6 proteins such as PLAUR function, e.g., in cellular adhesion [24]. *LYPD6* overexpression can inhibit transcriptional activity of the AP1 transcription factor complex [24], a key inflammation mediator activated, e.g., in endothelial cells in atherogenic disturbed blood flow conditions, leading in turn to upregulation of pro-inflammatory molecules [25]. Similar transcriptional changes have been found in the ruptured human sIA wall [26]. *MMADHC* is an intracellular vitamin B12 trafficking gene. Mutations in this gene can cause methylmalonic aciduria or homocystinuria, or both [27].

### 5q31.1 locus

The variant rs113816216 at 5q31.3 has a frequency of 1–3% in Finland and most other European populations, except in Spain (7%). It was significantly associated to the sIA disease in the Finnish samples and was also significant in the Dutch sample but had a somewhat lower OR there (Table 2). The meta-analysis of all combined samples exceeded the genome wide significance threshold. The variant is located in the intron of *FSTL4* (Figure 2 B), a poorly characterized gene. *FSTL1*, a paralog of *FSTL4*, codes a protein inducing innate immunity as *TLR4* agonist [28]. Increased tissue levels of *FSTL1* were associated to the severity of heart failure [29] and to the coronary artery aneurysm formation in Kawasaki disease [30]. Variants in *FSTL4* were modestly associated to human ischemic stroke [31], and a variant 70 kb from *FSTL4* nominally to hypertension [32].

### 6q24.2 locus

The variant rs75018213 at 6q24.2 has similar frequencies (2%) in European populations, including Finns. It was significantly associated to the sIA disease in the Finnish samples and was also significant in the Dutch sample but had a somewhat lower OR there (Table 2). It is located in the intron of *EPM2A*. The LD spans over 300 kb downstream covering *FBXO30*, *LOC100507557*, *SHPRH* and *GRM1* (Figure 2 C). In the ENCODE data, rs75018213 is located in a *GATA2* transcription factor binding site RNA-seq peak. Homozygous deletions in the EPM2A gene result in progressive myoclonus epilepsy (PME) with Lafora bodies (OMIM 254780) [33]. No vascular anomalies have been reported in EPM2 deletion patients with a PME phenotype or their heterozygote parents. *EPM2A* encodes a phosphatase, which dephosphorylates glycogen, but it is likely that *EPM2A* has broader functions in regulation of glycogen biosynthesis, endoplasmic reticulum stress, autophagy, and possibly also cell cycle [34].

### 7p22.1 locus and the number of sIAs

The variant rs150927513 at 7p22.1 was significantly associated to sIA count per individual in the Finnish population (Table 1). Its frequency was 4.6% in the Finnish samples but only 0.3%, in the Dutch sample, in line with most European populations. This variant would therefore likely not have been identified if a sufficient number of Finnish individuals had not been included in the reference panel.

The variant is located in the intron of *RADIL* (Figure 2 D), a rap GTPase interactor, an essential effector of *RAP1* in activation of integrins in cell-adhesive signalling by G protein-coupled receptors [35]. *RADIL* has also been shown to control, together with *RAP1*, neutrophil adhesion and chemotaxis [36]. Neutrophils seem to have a role in the formation and rupture of intracranial and abdominal aortic aneurysm [26], [37], [38]. The strongest eQTL association was to an exon of *TNRC18* (FC 1.23, p = 5.1×10^−5^), a functionally uncharacterized gene.

As we analysed the number of sIAs as a count variable from 0–8, the inherent assumption was that the same variant would increase the risk of the first and the subsequent sIA formation. Thus, any variant associated to the number of sIAs will to some extent be associated in the case vs. control analysis. Indeed, in the analysis of combined Finnish cohorts rs150927513 was associated in the case-control analysis (OR 1.54, p = 6.5×10^−7^) and consistently also in the analysis of multiple vs. single sIA patients (OR 1.65, p = 8.4×10^−4^). The association of this variant, should be interpreted as reflecting the tendency of sIA formation, rather than considering multiple sIAs as a completely different dichotomous end point.

### Previously identified 9p21.3 locus

The 9p21.3 locus has been robustly associated to the sIA disease [12] as well as to cardiovascular, metabolic and cancer traits [39], [40], and it has been extensively studied by others [41]. The allele frequency and effect size in the current study, although with a different lead SNP (r^2^ = 0.7 to previous lead SNP rs1333040), are in strong agreement with the previous study [12]. This locus is not therefore discussed further here.

### 2q33.1 locus with previously inconclusive evidence

Two common variants, rs12472355 and rs919433 at 2q33.1 were significantly associated to the sIA disease in the Finnish and Dutch samples (Table 2), rs919433 intronic and rs12472355 upstream 30 kb from *ANKRD44* (Figure 2 E). The allele frequencies were somewhat higher in the Finnish samples (rs919433, 44%; rs12472355 43.7%) than in the Dutch samples (33.2%; 31%) or in the Japanese population according to 1000 Genomes Project (28.1%; 27.5%). In this locus, the risk allele was reversed in the Japanese cohort of the previous sIA GWA study [10]. *ANKRD44* is likely a subunit of protein phosphatase 6 [42] that functions, e.g., in cell cycle control [43] and in inhibition of *NF-κB* activation [44]. *NF-κB* is a significant mediator in experimental sIA formation in rats, highly expressed in human sIA wall [45], and it was associated to human sIA wall rupture in transcriptomic profiling [26]. In eQTL analysis rs12472355 was significantly associated to *ANKRD44* (FC 0.94, p = 1.83×10^−5^) and rs919433 to *HSPD1* (FC 0.94, p = 3.8×10^−5^)

In conclusion, we identified four novel loci associated to sIA disease and confirmed one additional locus with previously inconclusive evidence, together explaining 2.1% of the sIA heritability in Finland. Our data illustrates the utility of high-risk population isolates, familial disease history, and dense genotype imputation in search for low-frequency variants associated to complex human diseases. The inclusion of Finnish individuals in the imputation reference panel and especially the highly differentiated variant in 7p22.1 would likely not have been identified

The identification of the four novel low frequency variants would likely have required much larger sample sizes in more mixed populations. Further studies of the identified five loci are needed to explain their functional mechanisms in the pathogenesis of sIA disease.

## Materials and Methods

### Ethics statement

For all of the Finnish and Dutch samples, the local ethics committees approved the study and all patients gave written informed consent.

### Study samples

#### A. Finnish discovery sample

The initial discovery GWAS data consisted of previously Illumina genotyped 974 Finnish intracranial aneurysm patients and 740 controls [12]. The patients were collected from the registries of Neurosurgery, Kuopio University Hospital, and Neurosurgery, Helsinki University Hospital, solely serving their catchment populations in Eastern and Southern Finland, respectively. The sIAs were angiographically verified and the cases of subarachnoid hemorrhage from ruptured sIA (sIA-SAH) with computed tomography (CT). Patients with at least 1 first-degree relative carrying sIA disease were considered familial [8]. For the unruptured aneurysms we do not have the exact indications for these patients available. However in our aneurysm database in Neurosurgery of Kuopio University Hospital the indications for angiography of unruptured aneurysm patients were: 1) Incidental unruptured sIA (leading cause was headache) found in neuroimaging with non-related indications 383/467 = 83% 2) Incidential unruptured sIA found in neuroimaging screening of sIA family members 45/467 = 9.6% and 3) Symptomatic but unruptured sIA causing focal neurological symptoms 39/467 = 8.4%

The Helsinki Birth Cohort Study (HBCS) includes 8,760 individuals born in the Helsinki Central Hospital between 1934 and 1944 [46]. A subset of 1676 Illumina genotyped individuals were available for the present study. The Health 2000 Cohort (H2000) includes 2 402 Finns, and of those 2138 Illumina genotyped individuals were available for the present study [47], [48].

The discovery aneurysm cases, 740 population controls and Health 2000 controls have been used in the previous sIA GWA studies [10], [12].

The following 210 cases and 119 controls were removed from the discovery sample: fusiform aneurysm carriers (n = 5); duplicated cases (n = 9) and controls (n = 10); blind duplicate cases (n = 15) and controls (n = 5); genotyping rate <97% (29 cases, 31 controls); individuals with higher missingness from cryptically related pairs (Identity by descent (IBD)>0.1875, similarity halfway between 2nd and 3rd degree relatives: 69 cases, 55 controls); genetic distance to 5 nearest neighbours >4 standard deviations longer than the average distance (2 cases, 18 controls); patients not traceable from the database or with traumatic SAH (n = 81); polycystic kidney disease (n = 4).

The following SNPs were removed: missing genotypes >5%; minor allele frequency <1%; Hardy-Weinberg disequilibrium p-value in controls <1*10-6; symmetric SNPs (A/T, C/G); and SNPs not on all the genotyping platforms.

To minimize false positives, each sIA case was matched to three controls by gender and genetic distance from control individuals. First, a sliding window approach was used to thin the set of SNPs to be approximately independent of each other. A sliding window of 1500 SNPs was shifted by 150 SNPs at a time along chromosomes, and in each step SNPs were filtered if any pairwise r2 was >0.2, resulting in 79596 independent SNPs. Pairwise IBS distances of these SNPs were used in multidimensional scaling and four first dimensions were used in matching. Plink v. 1.07 [49] was used for thinning and MDS analysis. R package optmatch was used to pair each case to three controls. After 1∶3 matching, additionally all Eastern Finnish controls from the previous sIA study were included [12].

The final discovery sample consisted of 760 sIA cases and 2,513 controls (Table 1). After SNP filtering, there were 304,399 genotyped SNPs and 9,046,433 imputed SNPs and indels (see imputation paragraph for imputation QC) for the discovery sample.

#### B. Finnish replication sample

The replication sample consisted of 858 independent sIA patients from the registry of Neurosurgery, Kuopio University Hospital. There were 1,605 independent controls, 453 from Eastern Finland and 1152 from the FINRISK study, both genotyped using the Sequenom iPLEX technique. Additionally, 2,443 whole genome genotyped controls from The Cardiovascular Risk in Young Finns Study were acquired and replication SNPs were extracted after imputation (Table 1).

The Cardiovascular Risk in Young Finns Study is a follow-up study of cardiovascular risk factors from childhood to adulthood [50], [51]. The participants were randomly chosen from the Finnish Population Registry and recruited from five university cities in Finland. The baseline study launched in 1980 and included 3,596 individuals. Follow-ups have taken place at every three to six years with the last one in 2007 at 27 years of age.

The FINRISK cohort is a national survey on risk factors of chronic and non-communicable diseases in Finland [52]. The survey has been conducted every five years since 1972 in randomly selected, representative population samples from different parts of Finland.

#### C. Dutch replication sample

The Dutch sample consisted of previously GWAS genotyped 786 Dutch sIA cases (Yasuno 2010), and the 3,110 controls were recruited as part of the Nijmegen Biomedical Study (n = 1,832) and the Nijmegen Bladder Cancer Study (n = 1,278) [53], [54]. The relevant medical ethical committees approved all studies and all participants provided written informed consent.

The patients were admitted to the Utrecht University Medical Center between 1997 and 2007. The sIA-SAH cases were verified with CT scan and sIAs by angiography. Unruptured sIAs were identified by angiography in the absence of clinical or radiological signs of SAH [12]. Patients reporting at least 1 first-degree relative carrying sIA disease were considered familial.

The Nijmegen Biomedical Study is a population based cross-sectional study conducted by the Radboud University Nijmegen Medical Centre [53], [54]. Age and sex stratified, randomly selected adults (≥18 years) of Nijmegen (n = 22,452) received an invitation to fill out a postal questionnaire on lifestyle and medical history.

The following cases and controls were excluded: missingness ≥0.05 (n = 10); IBD≥0.2 (n = 102); heterozygosity >/<3 standard deviations from the mean (n = 46); and principal component analysis outliers (n = 43). The intersection of SNPs in different platforms was first extracted and symmetric SNPs were removed (A/T, C/G). SNPs prior to the imputation were filtered by the following QC criteria: genotype missingness >0.05; MAF<0.01; HWE p<0.001; differential missingness between cases and controls p<1E-5.

The final Dutch replication sample consisted of 717 cases and 3,004 controls (Table 1).

### Replication strategy

From both of the analyses (the case vs. controls and the number of sIAs) the best independent SNPs were taken to replication if p<5E-6. Additional significant independent SNPs in a locus was tested by analyzing each SNP within 1 MB from the top SNP while adding the top SNP as a covariate. Additionally the most significant SNP in the current study in 2q33.1 region with uncertain evidence in previous sIA GWASs was taken to replication. Variant was considered replicated if it reached one-tailed significance of p<0.05 and was consistent in terms of risk allele. In all of the results, one-tailed p-values are given for the Finnish replication and in Dutch results.

### Genotyping

Genomic DNA was extracted from peripheral blood and genotyped by Illumina arrays: the Finnish discovery sample and the Dutch replication cases by CNV370k DUO chip; the HBCS and YFS controls by Illumina Human670K customBeadChip; and the H2000 controls by Illumina Infinium HDHuman610-Quad BeadChip.

In the Finnish replication sample, DNA was genotyped using Sequenom MassARRAY system and iPLEX Gold assays (Sequenom Inc., San Diego, USA). The data was collected using the MassARRAY Compact System (Sequenom) and the genotypes were called using TyperAnalyzer software (Sequenom). Genotyping quality was examined by a detailed QC procedure consisting of success rate checks, duplicates, water controls and Hardy-Weinberg Equilibrium (HWE) testing. SNPs were filtered if genotype missingness >0.05 or if HWE p<0.001.

### Imputation

For imputation of additional genotypes in the discovery sample, the Young Finns replication cohort and in the 2^nd^ Dutch replication sample the genotypes were first pre-phased [55] using the Shape-IT [56] phasing software and the pre-phased haplotypes were subjected to imputation. The Impute version 2.2.2 software [57] with 1,000 Genomes Phase I integrated variant set release (v3) reference panel (05 Mar 2012 release downloaded from http://mathgen.stats.ox.ac.uk/impute/data_download_1000G_phase1_integrated.html) was used. Imputed genotypes were filtered if the Impute info measure was <0.5 or minor allele frequency <0.01 in the Finnish discovery sample.

### eQTL analysis

We analyzed whether the identified genome-wide significant SNPs might affect gene expression by using the European samples of the Geuvadis RNA-sequencing data set, with mRNA sequencing data from LCLs of 373 samples from the FIN, CEU, GBR and TSI populations of 1000 Genomes project (for details, see [21]).

We did eQTL analysis for each of the associating variants and all the genes within a 1 MB window that were expressed in >50% of the individuals (Table eQTL). We used exon quantifications based on individual read counts per exon, after correction by the total number of mapped reads per sample and PEER normalization to remove technical variation. For each exon, we calculated linear regression between these expression values and genotype dosage of the associating variants in the 1000 Genomes data.

### Regional association plots

Regional association plots were generated using LocusZoom with LD data from European populations of 1000 Genomes project (Hg19/March 2012) [58].

### Search of regulatory elements at identified variants

The UCSC Genome Browser and HaploReg version 2 [19] were used to search for ENCODE regulatory element regions located at the five genome-wide significant variants. HaploReg database also annotates if SNP resides on a putative transcription-factor binding site (TFBS) according to Transfac or Jaspar TFBS profiles and also 10 most enriched TFBS profiles identified in ENCODE TF Chip-Seq peaks. We used all the Jaspar and Transfac annotations and three most enriched ENCODE based TFBS annotations for each TF.

### Statistical analysis

GWA was performed against two complementary phenotypes: the case vs. control status and the number of sIAs.

#### Case vs. control analysis

SNPTEST v2.3.0 was used for the association analysis, assuming additive effect. Genotype uncertainty in the imputed SNPs was taken in to account by treating them as continuous expected genotype dosages. The gender was used as a covariate.

#### Aneurysm count analysis

The Vuong test [59] showed that the negative binomial model was a significantly better fit to the sIA count per individual when compared to the Poisson model. The zero-inflated negative binomial model was not significantly better either, so the simpler negative binomial model (glm.nb function in MASS R package) was used. When assessing the model fits, the gender was used as a predictor. Imputation uncertainty was taken in to account by treating the imputed SNPs as continuous expected genotype dosages, and the gender was used as a covariate.

#### Meta-analysis

The association evidence from the discovery and replication samples were combined by inverse variance-weighted fixed-effects meta-analysis, using Plink v.1.07 [49]. Heterogeneity statistic I2 and confidence intervals were calculated according to Higgins et al. [60] using metafor R package [61].

#### Genome-wide significance level estimation

As the standard genome-wide significance value of 5 * 10^−8^ is estimated to correct for independent tests when testing all common variants (MAF> = 5%). As we tested variants with MAF> = 1%, the standard genome-wide significance may be liberal. A simple Bonferroni correction would be much too string because of correlation between tested variants.

We estimated approximately independent number of variants by analysing chromosomes 1 and 7 of European individuals of the 1000 Genomes Project. We pruned the set of variants to be approximately independent (pairwise r2< = 0.6 within 250 kb of each other) using WDIST (https://www.cog-genomics.org/wdist/). This resulted in 308547 and 358834 independent variants out of 2215231 and 2553047 respectively. Taking the same proportion (14%) of SNPs from the 9 359 231 variants in the discovery is 1 303 594 variants which yields genome-wide significance of 3.82 * 10-8. We similarly estimated squared correlation r2 of the 528677 genotyped and imputed variants of all 3273 discovery samples in chromosome 7 using custom Python script. The proportion of approximately independent variants was 53 909 (10.2%), which is lower than in the full set of 1000 Genomes variants (threshold 5.2 * 10-8).

#### Heritability analysis

The fraction of additive genetic variance explained by the five identified loci was estimated using the liability threshold model [62]. The model assumes an additive effect at each locus, which shifts the mean of a normally distributed distribution of disease liability for each genotype. The combined genetic variance explained by the five SNPs (rs74972714, rs113816216, rs7501821, rs1509275133, rs12472355) in the five loci was assumed to be the sum of variances explained by each SNP. Risk allele frequencies in controls and OR's from combined Finnish samples was used and population prevalence of 3% of the sIA disease was assumed [1]. Heritability of the six previously identified lead SNPs (rs9298506, rs1333040, rs12413409, rs9315204, rs11661542, rs6841581) was estimated using the allele frequencies and effect sizes from the discovery cohort of the current study.

## Supporting Information

Figure S1 Quantile-quantile plot of case vs. control analysis.
(TIF)

Figure S2 Quantile-quantile plot of aneurysm count analysis.
(TIF)

Figure S3 Manhattan plot of case versus control analysis.
(TIFF)

Figure S4 Manhattan plot of aneurysm count analysis.
(TIFF)

Figure S5 Pairwise plot of eQTL association statistics vs. aneurysm association statistics in the discovery cohort. All variants within 1 MB of reported variants and with both eQTL and aneurysm data available are plotted. Nominal aneurysm association p-value threshold of p = 0.05 is shown as vertical line.
(TIF)

Figure S6 Regional eQTL association landscape of the five identified saccular intracranial aneurysm loci. The reported lead SNP association to sIA disease is shown as purple circle. All other data points are eQTL association p-values (only association p-values<0.001 are shown). Color coding indicates LD between the sIA variant and each eQTL variant. Association p-values (−log10 scale, y-axis) of variants are shown according to their chromosomal positions (x-axis). Blue lines indicate the genetic recombination rate (cM/Mb). Figures A–C present the loci identified in the case vs. control analysis at 2q23.3, 5q31.3, and 6q24.2, respectively. Figure D presents the 7p22.1 locus associated to the sIA count per patient. Figure E presents the 2q33.1 locus with inconclusive previous evidence.
(TIF)

Table S1 All variants analyzed in case vs. control analysis in the discovery and the replication phases.
(XLS)

Table S2 All variants analyzed in the aneurysm count analysis in the discovery and the replication phases.
(XLS)

Table S3 Genotype to aneurysm count distribution of genome-wide significant rs150927513 in combined Finnish discovery and replication cohorts.
(XLS)

Table S4 Regulatory elements at the identified variants.
(XLS)

Table S5 Previous GWAS studies of the sIA disease. Association results are reported according to chromosomal loci and differing SNP is indicated above each study column if different from primary study. Each cell reports [odds ratio; (pvalue); risk allele; allele frequency in controls] (e.g 1.6 (4.5E-4) 38%) unless otherwise noted.
(XLS)

Table S6 eQTL analysis results of correlating each genome-wide significant SNP to exon expression levels of genes < = 1 MB away from the index SNP.
(XLS)

Table S7 Imputation accuracy statistics of all genome-wide significant variants.
(XLS)

Table S8 Genotyping of 87 individuals of the discovery sample by direct genotyping.
(XLS)

Table S9 Heterogeneity statistics of meta-analysis combining all three samples.
(XLS)

Table S10 All variants with eQTL associations p<0.001 and aneurysm association (discovery sample) p<0.05 within 1 MB of reported variants.
(XLS)

Table S11 Validation of imputed genotypes by Sanger sequencing.
(XLS)

Table S12 Putative protein product function affecting variants within 1 MB of the identified variants in 583 whole exome sequenced Finnish individuals.
(XLS)

Table S13 In silico validation of genotype imputation accuracy in Dutch population using 96 individuals with both genotype chip data and high coverage(>40× on average) full genome sequencing data available.
(XLS)

Text S1 Description of simulation experiment to assess false positive probabilities due to imputation inaccuracy.
(DOCX)
