# Supplementary figures and images for: High Risk Population Isolate Reveals Low Frequency Variants Predisposing to Intracranial Aneurysms
Source: PLoS Genet. 2014 Jan 30;10(1):e1004134. doi: 10.1371/journal.pgen.1004134 (PMC3907358; doi:10.1371/journal.pgen.1004134)

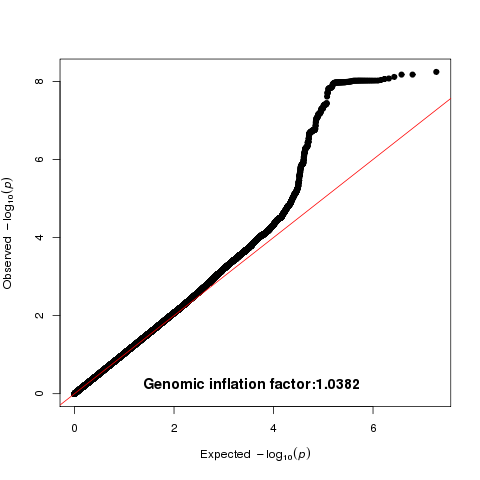

Supplement: Figure S1 — Quantile-quantile plot of case vs. control analysis. (TIF) [file pgen.1004134.s001.tif]

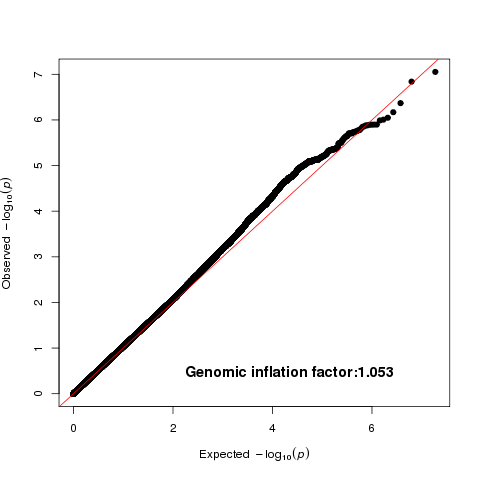

Supplement: Figure S2 — Quantile-quantile plot of aneurysm count analysis. (TIF) [file pgen.1004134.s002.tif]

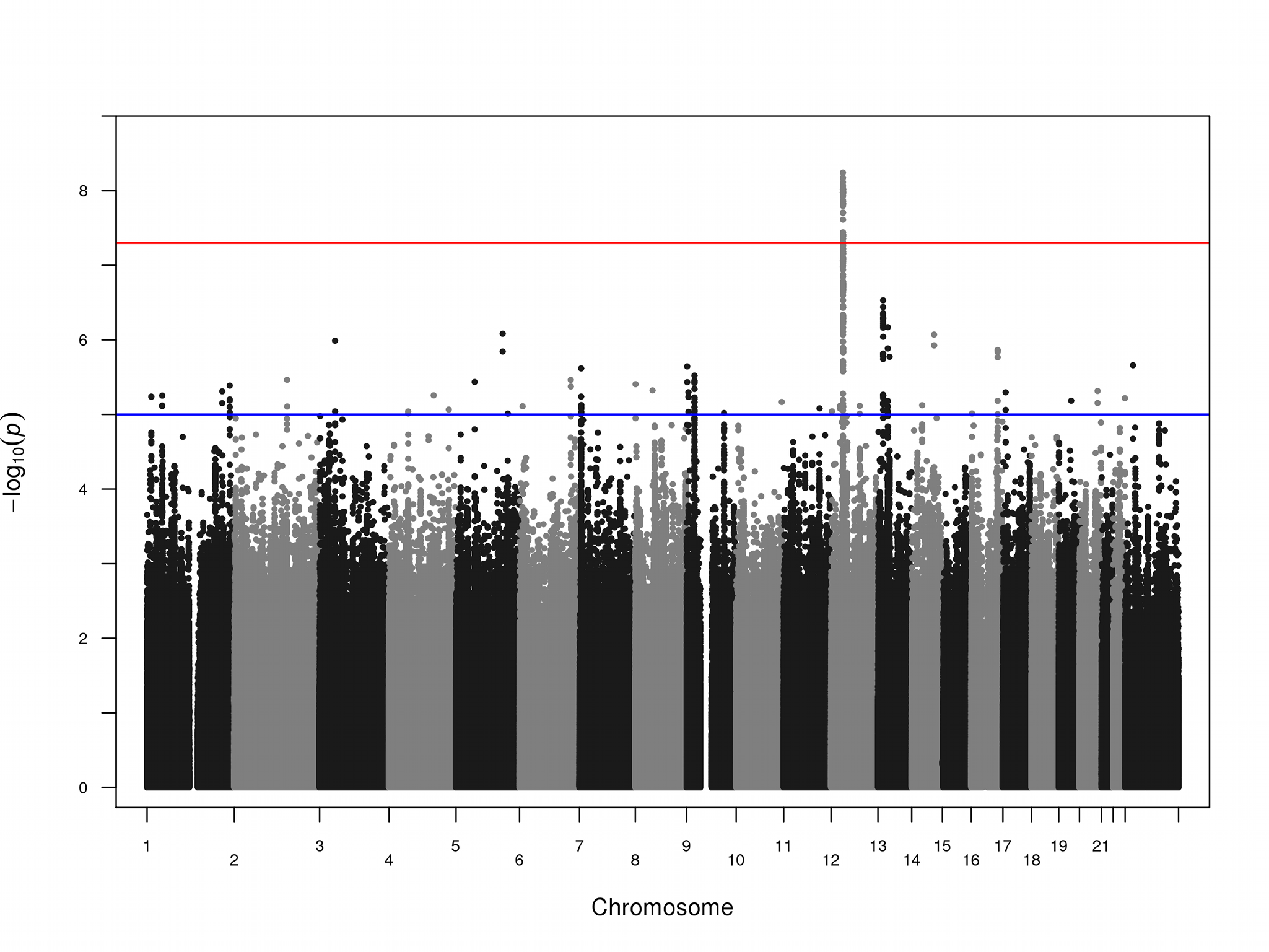

Supplement: Figure S3 — Manhattan plot of case versus control analysis. (TIFF) [file pgen.1004134.s003.tiff]

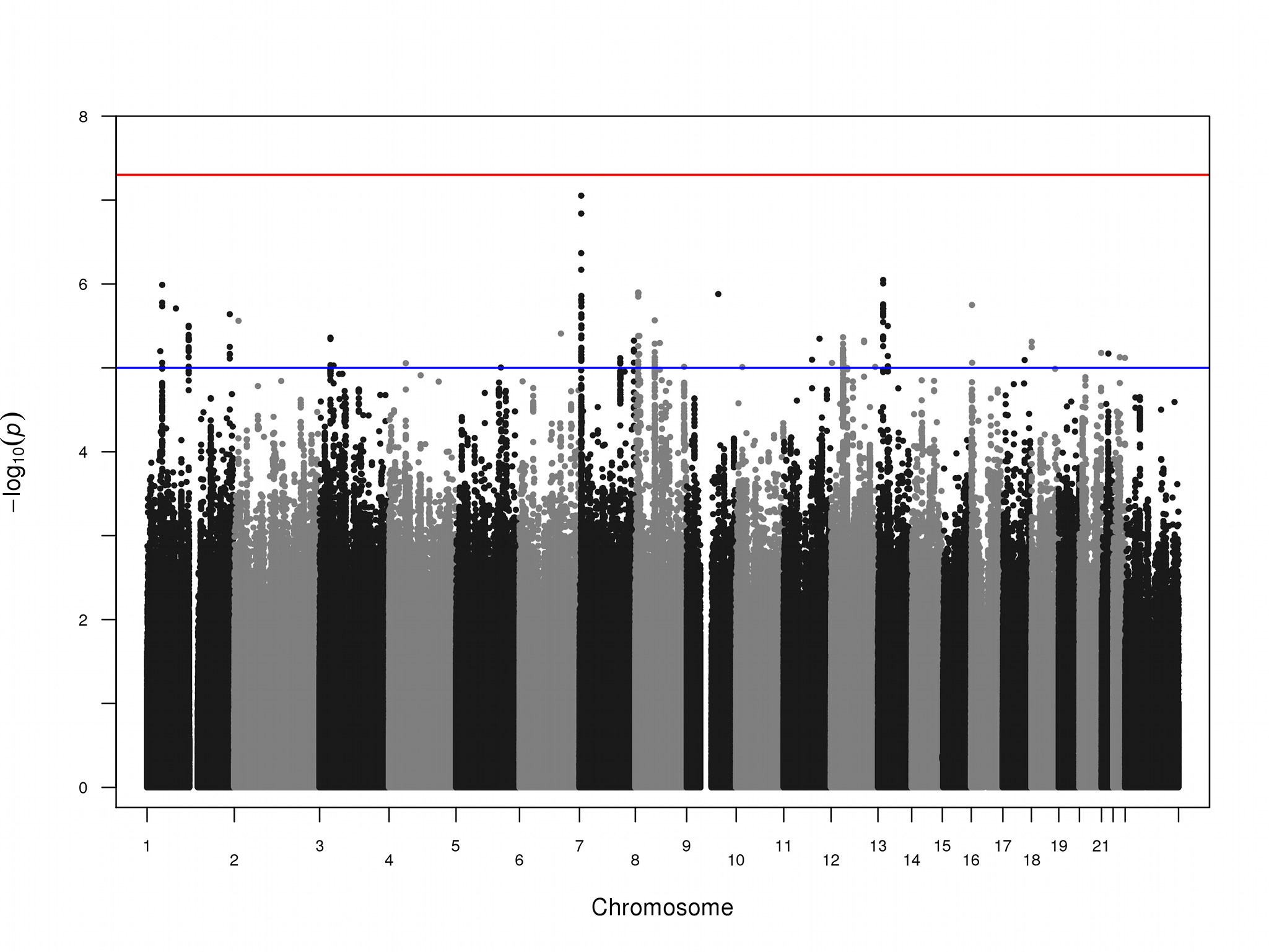

Supplement: Figure S4 — Manhattan plot of aneurysm count analysis. (TIFF) [file pgen.1004134.s004.tiff]

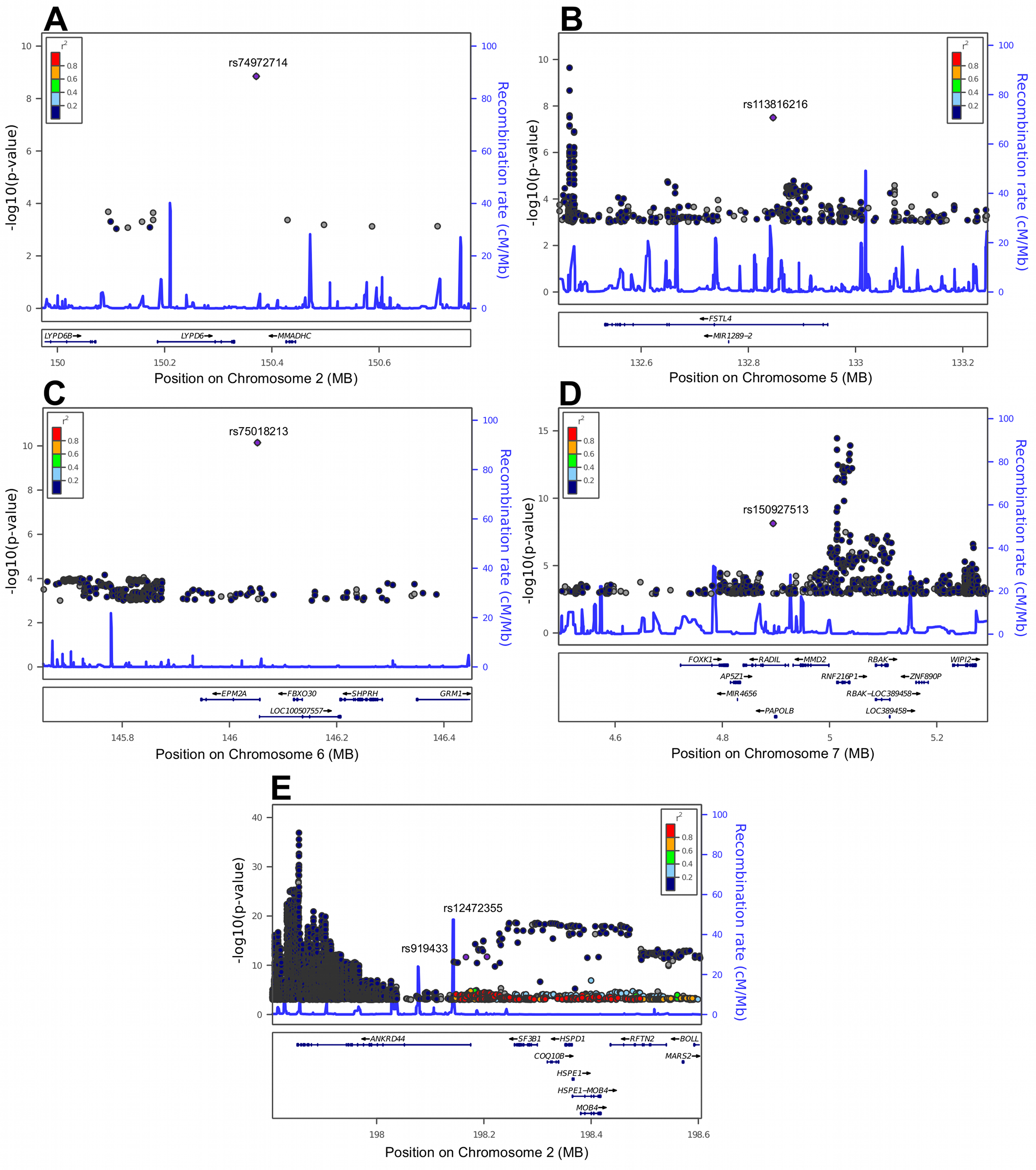

Supplement: Figure S6 — Regional eQTL association landscape of the five identified saccular intracranial aneurysm loci. The reported lead SNP association to sIA disease is shown as purple circle. All other data points are eQTL association p-values (only association p-values<0.001 are shown). Color coding indicates LD between the sIA variant and each eQTL variant. Association p-values (−log10 scale, y-axis) of variants are shown according to their chromosomal positions (x-axis). Blue lines indicate the genetic recombination rate (cM/Mb). Figures A–C present the loci identified in the case vs. control analysis at 2q23.3, 5q31.3, and 6q24.2, respectively. Figure D presents the 7p22.1 locus associated to the sIA count per patient. Figure E presents the 2q33.1 locus with inconclusive previous evidence. (TIF) [file pgen.1004134.s006.tif]
