# Supplementary material for: High Risk Population Isolate Reveals Low Frequency Variants Predisposing to Intracranial Aneurysms
Source: PLoS Genet. 2014 Jan 30;10(1):e1004134. doi: 10.1371/journal.pgen.1004134 (PMC3907358; doi:10.1371/journal.pgen.1004134)
Supplement: Text S1 — Description of simulation experiment to assess false positive probabilities due to imputation inaccuracy. (DOCX) [file pgen.1004134.s020.docx]

Supplementary Text 1

We assessed, by simulation, how likely it would be to get the results we observed for rs75018213 and rs74972714 in the discovery just by chance given the imputation accuracy. We first estimated conditional probability densities (kernel density estimation) for imputing genotype dosages 0-2 given the real genotype using the Sequenom replication data. We assumed that the MAF of rs75018213 and rs74972714 would be 1.6% (this is the MAF of Finns of 1000 Genomes project). We randomly sampled genotypes of 740 “cases” and 2513 “controls” from MAF 1.6% and then sampled imputed genotype dosages for each individual from the estimated probability densities. We did not have data on minor allele homozygotes so we could not estimate probability densities and we used twice the imputation dosage of heterozygotes for the rare cases of minor allele homozygotes.

We then performed case vs. control analysis of this imputed dataset. This process was repeated 10 000 times and the effect sizes for each simulation round were recorder. The proportion of times the effect size in simulations were larger than the effect actually observed was used as the estimate of chance finding. The probability of chance finding was very low (rs75018213 p: 0.0001 and rs74972714 p < 0.0001). We did further sensitivity analysis by allowing 20% over-imputation of minor allele for the cases in the simulations. Even allowing systematic bias towards over-imputation in cases, it is highly unlikely to get the observed OR just by chance (rs75018213 p: 0.005 and rs74972714 p: 0.0001). These simulations further support the view that the identified variant is not a false positive finding due to imputation inaccuracy.
